# Treatment strategies and survival of patients with connective tissue disease and pulmonary arterial hypertension: a COMPERA analysis

**DOI:** 10.1093/rheumatology/kead360

**Published:** 2023-07-18

**Authors:** Oliver Distler, Christian Ofner, Dörte Huscher, Suzana Jordan, Silvia Ulrich, Gerd Stähler, Ekkehard Grünig, Matthias Held, H Ardeschir Ghofrani, Martin Claussen, Tobias J Lange, Hans Klose, Stephan Rosenkranz, Anton Vonk-Noordegraaf, C Dario Vizza, Marion Delcroix, Christian Opitz, Christine Pausch, Laura Scelsi, Claus Neurohr, Karen M Olsson, J Gerry Coghlan, Michael Halank, Dirk Skowasch, Jürgen Behr, Katrin Milger, Bjoern Andrew Remppis, Andris Skride, Elena Jureviciene, Lina Gumbiene, Skaidrius Miliauskas, Judith Löffler-Ragg, Heinrike Wilkens, David Pittrow, Marius M Hoeper, Ralf Ewert

**Affiliations:** Department of Rheumatology, University Hospital Zurich, University of Zurich, Zurich, Switzerland; Department of Rheumatology, University Hospital Zurich, University of Zurich, Zurich, Switzerland; Institute of Biometry and Clinical Epidemiology, and Berlin Institute of Health, Charité-Universitätsmedizin, Corporate member of Freie Universität Berlin and Humboldt Universität zu Berlin, Berlin, Germany; Department of Rheumatology, University Hospital Zurich, University of Zurich, Zurich, Switzerland; Department of Pulmonology, University Hospital Zurich, University of Zurich, Zurich, Switzerland; Klinik für Pneumologie, Klinik Fachklinik Löwenstein, Löwenstein, Germany; Center for Pulmonary Hypertension, Thoraxklinik at Heidelberg University Hospital, Translational Lung Research Center Heidelberg (TLRC), Member of the German Center for Lung Research, Heidelberg, Germany; Department of Internal Medicine, Respiratory Medicine and Ventilatory Support, Medical Mission Hospital, Central Clinic Würzburg, Würzburg, Germany; Department of Internal Medicine, Justus-Liebig-University Giessen, Universities of Giessen and Marburg Lung Center, Giessen, Germany; Fachabteilung Pneumologie, LungenClinic Großhansdorf, Großhansdorf, Germany; Department of Internal Medicine II, University Medical Center Regensburg, Regensburg, Germany; Department of Respiratory Medicine, Eppendorf University Hospital, Hamburg, Germany; Clinic III for Internal Medicine (Cardiology) and Center for Molecular Medicine and the Cologne Cardiovascular Research Center, University of Cologne, Cologne, Germany; Department of Pulmonary Medicine, Amsterdam UMC, Vrije Universiteit Amsterdam, Amsterdam Cardiovascular Sciences, Amsterdam, The Netherlands; Dipartimento di Scienze Cliniche Internistiche, Anestiologiche e Cardiolohiche, Sapienza, University of Rome, Rome, Italy; Clinical Department of Respiratory Diseases, University Hospitals of Leuven and Laboratory of Respiratory Diseases and Thoracic Surgery (BREATHE), Department of Chronic Diseases and Metabolism, KU Leuven–University of Leuven, Leuven, Belgium; Department of Cardiology, DRK Kliniken Berlin Westend, Berlin, Germany; GWT-TUD GmbH, Innovation Center Real World Evidence, Dresden, Germany; Fondazione IRCSS S. Matteo Pavia, Division of Cardiology Stolfo Davide, Azienda Sanitaria Universitaria Giuliano Isontina, Pavia, Italy; Department of Pulmonology and Respiratory Medicine, Robert-Bosch-Krankenhaus Stuttgart, Stuttgart, Germany; Department of Respiratory Medicine, Hannover Medical School, Hannover, Germany; German Center of Lung Research, Gießen, Germany; Department of Cardiology, Royal Free Hospital, London, UK; Division of Pulmonology, Medical Department I, University Hospital Carl Gustav Carus of Technical University Dresden, Dresden, Germany; Innere Medizin–Kardiologie/Pneumologie, Medizinische Klinik und Poliklinik II, Universitätsklinikum Bonn, Bonn, Germany; Department of Medicine V, University Hospital, LMU Munich, Comprehensive Pneumology Center Munich, Member of the German Center for Lung Research, Munich, Germany; Department of Medicine V, University Hospital, LMU Munich, Comprehensive Pneumology Center Munich, Member of the German Center for Lung Research, Munich, Germany; Herz- und Gefäßzentrum Bad Bevensen, Bad Bevensen, Germany; VSIA Pauls Stradins Clinical University Hospital, Riga, Lativa; Faculty of Medicine of Vilnius University, Competence Centre of Pulmonary Hypertension, Vilnius University Hospital Santaros klinikos, Vilnius, Lithuania; Faculty of Medicine of Vilnius University, Competence Centre of Pulmonary Hypertension, Vilnius University Hospital Santaros klinikos, Vilnius, Lithuania; Department of Pulmonology, Lithuanian University of Health Sciences, Kaunas, Lithuania; Department of Internal Medicine II, Medical University of Innsbruck, Innsbruck, Austria; Innere Medizin V, Universitätsklinikum des Saarlandes, Homburg, Germany; GWT-TUD GmbH, Innovation Center Real World Evidence, Dresden, Germany; Institute for Clinical Pharmacology, Medical Faculty, Technical University, Dresden, Germany; Department of Respiratory Medicine, Hannover Medical School, Hannover, Germany; German Center of Lung Research, Gießen, Germany; Clinic of Internal Medicine, Department of Respiratory Medicine, Universitätsmedizin Greifswald, Germany

**Keywords:** pulmonary arterial hypertension, connective tissue disease (CTD), systemic sclerosis (SSc), endothelin receptor antagonists (ERA), phosphodiesterase type 5 inhibitor (PDE5i)

## Abstract

**Objectives:**

Pulmonary arterial hypertension (PAH) occurs in various connective tissue diseases (CTDs). We sought to assess contemporary treatment patterns and survival of patients with various forms of CTD-PAH.

**Methods:**

We analysed data from COMPERA, a European pulmonary hypertension registry, to describe treatment strategies and survival in patients with newly diagnosed PAH associated with SSc, SLE, MCTD, UCTD and other types of CTD. All-cause mortality was analysed according to the underlying CTD. For patients with SSc-PAH, we also assessed survival according to initial therapy with endothelin receptor antagonists (ERAs), phosphodiesterase type 5 inhibitors (PDE5is) or a combination of these two drug classes.

**Results:**

This analysis included 607 patients with CTD-PAH. Survival estimates at 1, 3 and 5 years for SSc-PAH (*n* = 390) were 85%, 59% and 42%; for SLE-PAH (*n* = 34) they were 97%, 77% and 61%; for MCTD-PAH (*n* = 33) they were 97%, 70% and 59%; for UCTD-PAH (*n* = 60) they were 88%, 67% and 52%; and for other CTD-PAH (*n* = 90) they were 92%, 69% and 55%, respectively. After multivariable adjustment, the survival of patients with SSc-PAH was significantly worse compared with the other conditions (*P* = 0.001). In these patients, the survival estimates were significantly better with initial ERA–PDE5i combination therapy than with initial ERA or PDE5i monotherapy (*P* = 0.016 and *P* = 0.012, respectively).

**Conclusions:**

Mortality remains high in patients with CTD-PAH, especially for patients with SSc-PAH. However, for patients with SSc-PAH, our results suggest that long-term survival may be improved with initial ERA–PDE5i combination therapy compared with initial monotherapy.

Rheumatology key messages

- Among patients with CTD-PAH, those with SSc had the highest mortality risk.
- Mortality risk was lowest in SLE-PAH, with MCTD, UCTD and other CTDs in between.
- In patients with SSc-PAH, survival was better with initial ERA–PDE5i combination therapy than with ERA or PDE5i monotherapy.

## Introduction

CTDs are a diverse and heterogeneous group of rheumatic diseases that share common pathogenic pathways such as autoimmunity, inflammation, fibrosis and endothelial dysfunction [1]. One of the most meaningful disease manifestations is pulmonary arterial hypertension (PAH), caused by a progressive obliterative pulmonary vasculopathy [2]. PAH adds significantly to the disease burden of CTD patients and is one of the leading causes of death in patients with SSc [3]. The prevalence of PAH among the different CTDs varies substantially [4–7]. The prevalence of PAH among patients with SSc in western populations ranges between 7.5% and 12% [8–10]. The prevalence of PAH in patients with other CTDs is less well known.

Recent clinical trials and cohort studies have shown beneficial effects of upfront combination therapy of endothelin receptor antagonists (ERAs) and phosphodiesterase type 5 inhibitors (PDE5is) in patients with various forms of PAH, including CTD-PAH [11, 12]. However, in clinical practice, many patients continue to receive initial monotherapy with either an ERA or a PDE5i [13]. Moreover, the concept of comparable effects between these two drug classes in SSc-PAH has been challenged by a retrospective observational study showing a longer time to clinical worsening with PDE5is than ERAs [14].

Registries have become an important source of real-life data, especially in rare diseases. The Comparative, Prospective Registry of Newly Initiated Therapies for Pulmonary Hypertension (COMPERA registry; clinicaltrials.gov NCT01347216) enrols patients with newly initiated treatment for all forms of pulmonary hypertension (PH) from >60 PH centres from 12 European countries. The combination of mandatory right heat catheterization (RHC) for the diagnosis of PAH together with well-defined classification criteria for the different CTDs is unique and provides an excellent opportunity to study these rare diseases.

In this study we assessed the COMPERA database to compare the characteristics and survival of patients with CTD-PAH according to the underlying condition and to evaluate in patients with SSc-PAH the long-term survival according to initial treatment regimens with ERA monotherapy, PDE5i monotherapy or combination therapy of both drug classes.

## Methods

### Setting

Patients enrolled into the COMPERA registry between 1 January 2009 and 1 March 2022 were included in the current analysis. The collected data consisted of demographic data, aetiology and subtype of PH according to the Dana Point classification, haemodynamics as measured by RHC, biomarkers, 6 min walk distance (6MWD), World Health Organization functional class (WHO-FC), pulmonary function test data and therapy regimen. Follow-up visits were recorded every 6 months and whenever the patient had clinical worsening, therapy-related serious adverse events, PAH-associated hospitalization, change in PAH therapy or died.

The study complied with the Declaration of Helsinki. The COMPERA registry was approved by the institutional review boards of all participating centres. Every patient signed an informed consent prior to enrolment.

### Inclusion and exclusion criteria

**Figure 1.**
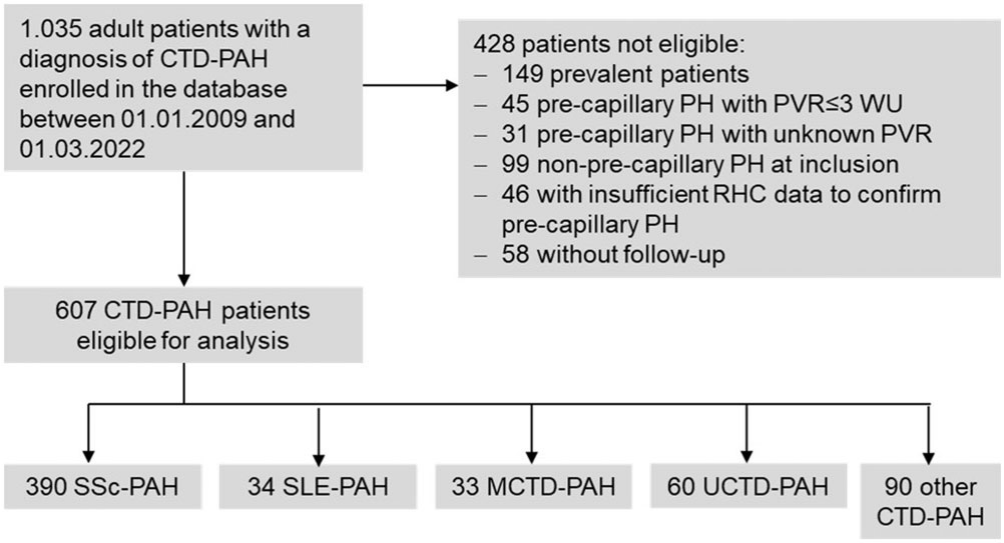
Flow chart of the selection process for the study cohort

CTD-PAH patients diagnosed with SSc, SLE, MCTD (anti-U1-RNP positive) and UCTD (not fulfilling any classification criteria, but evidence for autoimmune rheumatic disease) or other autoimmune rheumatic diseases, including e.g. SS, inflammatory myopathy, RA and overlap syndromes (fulfilling two classification criteria), were included. Inclusion was restricted to incident patients within 6 months of PAH diagnosis. The diagnosis of PAH had to be confirmed by RHC showing a mean pulmonary arterial pressure (mPAP) ≥25 mmHg, a pulmonary arterial wedge pressure (PAWP) of ≤15 mmHg and pulmonary vascular resistance (PVR) >3 Wood units. Juvenile patients, those having post-capillary PH or missing data about RHC and those without follow-up information were excluded from this analysis (Fig. 1). Three SSc-PAH patients receiving bosentan prior to inclusion for CTD-related indications other than PAH were excluded from the analysis of therapy effects.

### Study endpoints

The study endpoints were the comparison of 1-, 3- and 5-year survival between PAH patients with SSc, SLE, MCTD, UCTD and other CTD patients as well as survival of SSc-PAH patients according to their initial PAH treatment at baseline (ERA or PDE5i monotherapy or initial ERA–PDE5i combination therapy). Among all CTD patients, 15.2% changed therapy within 3 months after baseline. Among them, 26.1% changed from one monotherapy to a different one and 73.9% changed to combination therapy. Changes in PAH medications during the course of the disease were not considered.

### Data collection and statistical analysis

Patient data for this study were extracted from the COMPERA database on 1 March 2022. Data are shown as frequency with percentages, mean (s.d.) or median and interquartile range (IQR). For continuous data, group differences were compared by the Student’s *t*-test in case of normal distribution or by the Mann–Whitney *U* test otherwise. Frequency differences were compared by chi-squared or Fisher’s exact test. Multiple group-wise comparisons were performed with post hoc adjustment for the respective number of parallel tests; WHO-FC categories I and II were merged for testing. Survival was evaluated with Kaplan–Meier analysis and the Breslow test; observation times were censored at 5 years. Differences in survival between the CTD subtypes were investigated with Cox regression analysis with backward selection; missing values were imputed with fully conditional specification (FCS), an iterative Markov Chain Monte Carlo (MCMC) method appropriate for data with an arbitrary (non)monotone missing pattern with 10 repetitions. All variables selected in >50% of the 10 imputed data sets were retested in a Cox regression model without selection; pooled results are reported, which can turn non-significant for single variables of the prior selection process. *P*-values <0.05 were considered significant. SPSS Statistics version 24.0 (IBM, Armonk, NY, USA) was used for analysis.

## Results

At the time of data extraction, 11 188 consecutive patients with PH were enrolled in the COMPERA registry. Of those, 1035 patients had a CTD diagnosis. The selection process for the current study is shown in Fig. 1. After the exclusion of 428 patients not fulfilling the inclusion criteria for this study, a total of 607 patients with incident CTD-PAH, confirmed by RHC, remained for the present analysis. Underlying CTDs were SSc (*n* = 390), UCTD (*n* = 60), SLE (*n* = 34), MCTD (*n* = 33) and others (*n* = 90), which consisted mainly of RA (30%), SS (24%), overlap (13%) or inflammatory myopathies (13%) (Supplementary Table S1, available at *Rheumatology* online).

### Comparison according to the underlying condition

#### Baseline characteristics and initial treatment strategy

The baseline characteristics of the different CTD-PAH groups are shown in Table 1. Patients with SLE-PAH and MCTD-PAH were significantly younger than patients with SSc-PAH, UCTD-PAH and other CTD-PAH (all *P* < 0.001). Patients with SLE-PAH had a higher mPAP than patients with SSc-PAH (*P* = 0.035) and a higher PVR than patients with SSc-PAH (*P* = 0.006), UCTD-PAH (*P* = 0.015) and other CTD-PAH (*P* = 0.033). Regarding lung function, SSc-PAH patients had a higher FVC (*P* = 0.045) and a higher forced expiratory volume in 1 sec (FEV_1_; *P* = 0.048) than SLE-PAH. Other than that, there were no significant differences between the five groups.

**Table 1.** Characteristics at enrolment and initial therapy in patients with CTD-PAH

Characteristics	SSc (*n* = 390)	SLE (*n* = 34)	MCTD (*n* = 33)	UCTD (*n* = 60)	Other CTD (*n* = 90)
Age, years	68 (11)	51 (18)	57 (17)	70 (11)	68 (14)
Female, *n* (%)	321 (82.3)	27 (79.4)	29 (87.9)	42 (70.0)	65 (72.2)
WHO-FC, *n* (%) (*n* = 574)					
I	1 (0)	0 (0)	0 (0)	0 (0)	1 (1)
II	48 (13)	3 (9)	3 (9)	10 (17)	10 (11)
III	273 (75)	26 (79)	25 (78)	43 (74)	64 (73)
IV	41 (11)	4 (12)	4 (13)	5 (9)	13 (15)
6-MWD, m, median (IQR) (*n* = 455)	280 (180–380)	274 (232–350)	311 (213–418)	264 (170–350)	275 (180–367)
SvO_2_, % (*n* = 495)	64 (9)	60 (9)	64 (9)	61 (11)	63 (8)
mPAP, mmHg	41 (11)	47 (13)	42 (11)	43 (11)	42 (13)
PVR, Wood units	8.6 (4.1)	11.2 (6.0)	9.1 (5.6)	8.3 (3.9)	8.7 (4.0)
PAWP, mmHg	9 (3)	10 (3)	8 (3)	9 (4)	9 (3)
RAP, mmHg (*n* = 539)	8 (5)	9 (5)	7 (5)	9 (6)	8 (4)
Cardiac index, l/min/m^2^ (*n* = 556)	2.4 (0.7)	2.2 (0.7)	2.5 (0.7)	2.4 (0.8)	2.2 (0.6)
TLC, % predicted (*n* = 455)	87 (22)	81 (21)	85 (19)	81 (18)	84 (18)
FVC, % predicted (*n* = 493)	84 (24)	72 (22)	74 (20)	77 (20)	76 (20)
FEV_1_, % predicted (*n* = 501)	80 (21)	70 (21)	72 (18)	75 (20)	75 (21)
DLCO, % predicted (*n* = 412)	39 (15)	46 (19)	47 (14)	45 (19)	42 (17)
NT-proBNP, pg/ml, median (IQR) (*n* = 410)	1612 (431–3939)	2550 (1298–6259)	2144 (960–3126)	1630 (713–3116)	2160 (1063–5474)
ERA monotherapy, *n* (%)	124 (32)	6 (18)	10 (30)	11 (18)	16 (18)
PDE5i monotherapy, *n* (%)	147 (38)	15 (44)	16 (49)	38 (63)	58 (64)
ERA–PDE5i dual therapy, *n* (%)	82 (21)	9 (27)	5 (15)	6 (10)	9 (10)

Values are presented as mean (s.d.) unless stated otherwise. In case of missingness, the number of available values is shown.

SvO_2_: mixed venous oxygen saturation; RAP: right atrial pressure; TLC: total lung capacity; NT-proBNP: N-terminal prohormone of brain natriuretic peptide.

Irrespective of the underlying condition, the majority of patients received initial monotherapy with an ERA or a PDE5i; the proportion of patients receiving initial ERA–PDE5i combination therapy ranged from 10 to 27% (Table 1).

#### Survival

Survival status at the last available visit was known for 96.4% of all patients with a mean follow-up time of 32, 39, 42, 37 and 42 months for SSc-PAH, SLE-PAH, MCTD-PAH, UCTD-PAH and other CTD-PAH, respectively.

During the first 5 years after RHC diagnosis and initiation of PH therapy, 158 deaths occurred in patients with SSc-PAH, 8 in patients with SLE-PAH, 9 in patients with MCTD-PAH, 22 in patients with UCTD and 31 in patients with other CTD-PAH. In all CTD subgroups, the leading cause of death was right heart failure [SSc-PAH, *n* = 75 (47.5%); SLE-PAH, *n* = 3 (37.5%); MCTD-PAH, *n* = 7 (77.8%); UCTD-PAH, *n* = 6 (27.3%) and other CTD-PAH, *n* = 15 (48.4%)]. A detailed list of the causes of death is provided in Supplementary Table S2, available at *Rheumatology* online.

**Figure 2.**
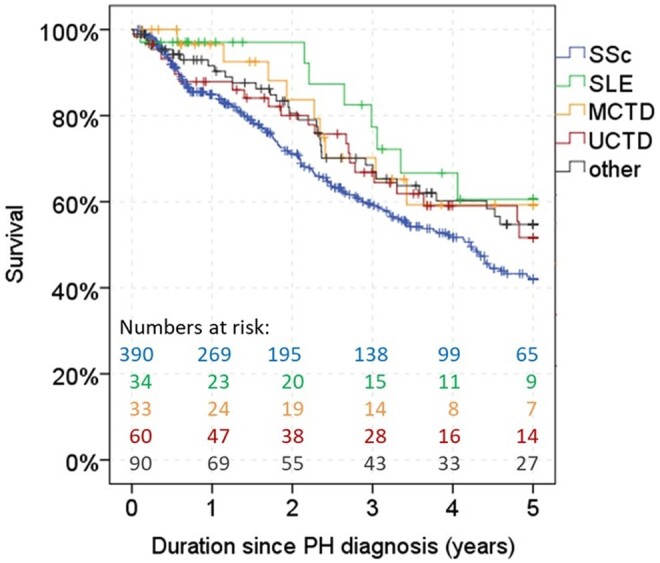
Kaplan–Meier survival estimates in various forms of CTD-PAH

The estimated 1-, 3- and 5-year survival by Kaplan–Meier was worst for SSc-PAH with 84.9%, 59.1% and 42.0%; best for SLE-PAH with 97.1%, 77.3% and 60.6% and MCTD-PAH with 96.6%, 70.2% and 59.3%; and intermediate for UCTD-PAH and other CTD-PAH with 87.9%, 66.8%, 51.7% and 91.6%, 68.5% and 54.7%, respectively (Fig. 2).

The estimated mean survival time was 3.4 years (95% CI 3.2, 3.6) for patients with SSc-PAH, 4.1 (95% CI 3.6, 4.6) for SLE-PAH, 3.9 (95% CI 3.3, 4.5) for MCTD-PAH, 3.7 (95% CI 3.2, 4.2) for UCTD-PAH and 3.8 (95% CI 3.4, 4.1) for other CTD-PAH. By Breslow test (overall *P* = 0.058), statistically significant differences in survival were only seen between SSc-PAH and SLE-PAH (Fig. 2; SSc-PAH *vs* SLE-PAH *P* = 0.041, SSc-PAH *vs* other CTD-PAH *P* = 0.076, all other groupwise comparisons *P* > 0.1). However, after adjustment in a multivariable Cox regression model, the type of CTD with SSc as the reference group was a significant prognostic factor, mainly driven by other CTD and UCTD (Table 2). When merging the other four CTD groups and comparing to SSc-PAH in the same multivariable model, SSc-PAH had a significantly higher mortality risk [odds ratio (OR) 1.7 (95% CI 1.2, 2.2), *P* = 0.001].

**Table 2.** Factors associated with survival in multivariable Cox regression

Factors	OR (95% CI)	*P*-value
SSc (ref)		
SLE	0.60 (0.28, 1.32)	0.205
MCTD	0.63 (0.31, 1.25)	0.187
UCTD	0.67 (0.42, 1.06)	0.090
Other CTD	0.56 (0.37, 0.84)	0.005
Male	1.43 (1.03, 2.00)	0.035
Age (per 10 years)	1.31 (1.14, 1.49)	<0.001
WHO-FC class IV^a^	2.30 (1.53, 3.48)	<0.001
6-MWD (per 50 m)	0.90 (0.82, 0.98)	0.020
PAWP	0.94 (0.90, 0.98)	0.005
Cardiac index	1.19 (0.96, 1.47)	0.122
DLCO (per 10% predicted)	0.88 (0.77, 1.00)	0.051
Log_10_(NT-proBNP)	1.81 (1.33, 2.44)	<0.001

Shown are pooled results of those variables that were selected in >50% of the 10 imputed data sets.

a As WHO-FC III did not show differences to classes I–II, classes I–III were merged.

NT-proBNP: N-terminal prohormone of brain natriuretic peptide.

### Comparison of initial monotherapy with ERA or PDE5i *vs* ERA–PDE5i combination therapy in patients with SSc-PAH

#### Baseline characteristics

In patients with SSc-PAH, baseline data were comparable in all treatment groups (Table 3), except for mPAP which was higher in the dual-therapy group compared with both monotherapy groups (*P* = 0.042 and *P* = 0.010, respectively). In the ERA monotherapy group (*n* = 124), 9 (7%) patients were treated with sitaxentan, 51 (41%) with bosentan, 16 (13%) with ambrisentan and 48 (39%) with macitentan. In the PDE5i monotherapy group (*n* = 147), 69 (47%) patients were treated with sildenafil and 78 (53%) with tadalafil. In the dual-therapy group (*n* = 81), 24 (30%) patients received ambrisentan plus tadalafil, 20 (25%) macitentan plus tadalafil, 15 (19%) macitentan plus sildenafil, 10 (12%) ambrisentan plus sildenafil, 9 (11%) bosentan plus sildenafil and 3 (4%) bosentan plus tadalafil.

**Table 3.** Baseline characteristics of patients with SSc-PAH initially receiving ERA monotherapy, PDE5i monotherapy or ERA–PDE5i combination therapy

Characteristics	ERA (*n* = 124)	PDE5i (*n* = 147)	ERA–PDE5i (*n* = 81)
Age, years	68 (9)	69 (11)	66 (11)
Female, *n* (%)	99 (80)	123 (84)	67 (83)
WHO-FC, *n* (%) (*n* = 331)			
I	1 (1)	0 0	0 0
II	13 (11)	16 (11)	16 (22)
III	91 (80)	108 (75)	48 (66)
IV	9 (8)	20 (14)	9 (12)
6-MWD, m, median (IQR) (*n* = 251)	304 (165–402)	255 (180–363)	270 (206–360)
SvO_2_, % (*n* = 285)	64 (9)	64 (8)	62 (11)
mPAP, mmHg	40 (11)	40 (10)	44 (11)
PVR, WU	8.5 (4.4)	8.2 (3.8)	9.2 (4.0)
PAWP, mmHg	9 (4)	9 (3)	9 (3)
RAP, mmHg (*n* = 309)	7 (5)	8 (6)	8 (4)
Cardiac index, l/min/m^2^ (*n* = 317)	2.4 (0.8)	2.4 (0.6)	2.3 (0.6)
TLC, % pred. (*n* = 248)	86 (25)	89 (22)	85 (18)
FVC, % pred. (*n* = 275)	85 (25)	84 (24)	82 (24)
FEV_1_, % pred. (*n* = 280)	81 (20)	80 (21)	80 (24)
DL_CO_, % pred. (*n* = 232)	40 (15)	41 (15)	40 (14)
NT-proBNP, pg/ml (*n* = 249)	1783 (431–3411)	1903 (534–4313)	1068 (398–3171)

Values are presented as mean (s.d.) unless stated otherwise. In case of missingness, the number of available values is shown.

SvO_2_: mixed venous oxygen saturation; RAP: right atrial pressure; NT-proBNP: N-terminal prohormone of brain natriuretic peptide.

#### Survival of patients with SSc-PAH according to initial therapy

Survival status at the last available visit was known for 96.6% of the selected patients with SSc-PAH. The number of patients lost to follow-up for the groups with initial ERA monotherapy, PDE5i monotherapy and ERA–PDE5i combination therapy was five (4.0%), two (1.4%) and five (6.2%), respectively. Within 5 years after PAH diagnosis, 60 deaths occurred in patients with initial ERA monotherapy, 61 in patients with initial PDE5i monotherapy and 19 in patients with ERA–PDE5i dual therapy.

The leading cause of death in all three groups was right heart failure [*n* = 30 (50%) with ERA monotherapy, *n* = 28 (46%) with PDE5i monotherapy and *n* = 8 (42%) with ERA–PDE5i dual therapy]. A detailed list of the causes of death is provided in Supplementary Table S3, available at *Rheumatology* online.

**Figure 3.**
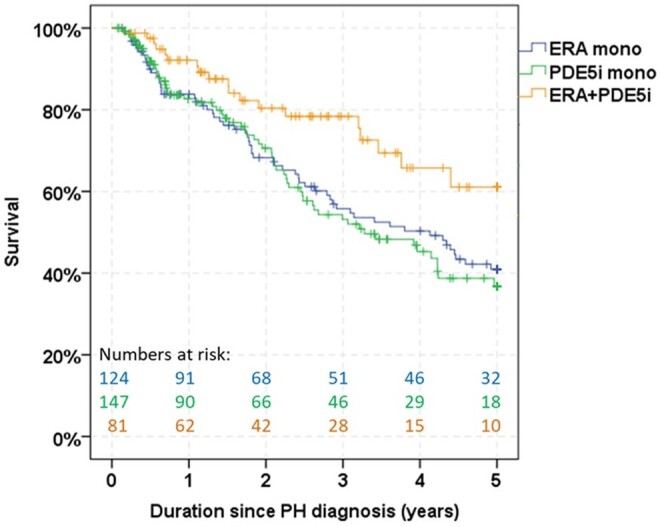
Kaplan–Meier survival estimates in patients with SSc-PAH according to the initial treatment regimen

For patients with initial ERA monotherapy, 1-, 3- and 5-year Kaplan–Meier survival estimates were 84%, 56% and 41%; for PDE5i monotherapy patients they were 83%, 53% and 37%; and for ERA–PDE5i combination therapy they were 92%, 78% and 61%, respectively. The mean survival time was 3.3 years (95% CI 2.9, 3.6) with ERA monotherapy, 3.2 years (95% CI 2.9, 3.5) with PDE5i monotherapy and 4.0 years (95% CI 3.6, 4.4) with ERA–PDE5i combination therapy. While no survival difference was seen between the ERA and the PDE5i monotherapy groups (*P* = 0.646), the survival in the ERA–PDE5i combination group was significantly better than in the monotherapy groups (ERA *vs* combination therapy, *P* = 0.016; PDE5i *vs* combination therapy, *P* = 0.012) (Fig. 3). This difference remained after multivariable adjustment for factors associated with survival (Supplementary Table S4, available at *Rheumatology* online).

## Discussion

This study reports on a large population of incident patients with various forms of CTD and PAH. The PAH diagnosis was confirmed by RHC in all patients. Irrespective of the underlying condition, CTD-PAH was associated with a high mortality risk, most notably in patients with SSc-PAH. In these patients, our data suggest that initial ERA–PDE5i combination therapy may confer a survival benefit over initial monotherapy with these compounds.

It is already known that patients with SSc-PAH have a particularly poor survival, especially when compared with patients with SLE-PAH [15–23] and MCTD-PAH [21]. This finding was confirmed by the present study despite patients with SLE-PAH presenting with the worst haemodynamic impairment at baseline. Moreover, to the best of our knowledge, it has not been shown before that the survival of patients with SSc-PAH is worse than that of patients with UCTD-PAH and PAH associated with other CTDs. It has to be noted that patients with SSc may also present with, in addition to PAH, group 3 PH (associated with hypoxia and lung disease) or pulmonary veno-occlusive disease. This is of importance, because these entities are characterized by worse prognosis and may contribute to the high mortality risk of SSc patients.

Comparing baseline characteristics at the time of PAH diagnosis, patients with SSc-PAH were older than patients with SLE-PAH and patients with MCTD-PAH, in accordance with studies from the USA and UK [16, 18]. Only marginal differences in haemodynamics and pulmonary function were noted between patients with SSc-PAH and patients with other CTDs, except for the diffusing capacity of the lung for carbon monoxide (DLCO), which was lowest in patients with SSc-PAH. Younger age and a higher DLCO may have contributed to the better survival of patients with MCTD-PAH and SLE-PAH compared with patients with SSc-PAH, but other reasons may apply as well that were not captured in the present analysis. For instance, left ventricular systolic and diastolic dysfunction as well as pulmonary fibrosis are more prevalent in SSc-PAH than in other forms of CTD-PAH [24, 25]. In addition, while no evidence about effectiveness of immunosuppressants in patients with SSc-PAH exists, patients with SLE-PAH and MCTD-PAH benefit from immunosuppression [26–28].

The optimal treatment of CTD-PAH remains unknown. The CTD subgroup analysis from Ambrisentan and Tadalafil in Patients with Pulmonary Arterial Hypertension (AMBITION) study showed that patients had a better treatment response, as determined by the time to treatment failure, with initial combination therapy than with PDE5i or ERA monotherapy [11, 12]. In contrast, a retrospective analysis of the Pulmonary Hypertension Assessment and Recognition of Outcomes in Scleroderma (PHAROS) registry, including 98 patients with SSc-PAH, fuelled the discussion over the optimal therapeutic approach in newly diagnosed SSc-PAH patients. The authors found that initial therapy with an ERA was associated with a significantly shorter time to clinical worsening when compared with an initial PDE5i treatment or a combination of a PDE5i and an ERA therapy [14]. In the present study we were unable to reproduce the PHAROS results, as we found no survival difference between the ERA and the PDE5i monotherapy groups, but there was a significant survival benefit for patients who received initial ERA–PDE5i combination therapy. This survival benefit is noteworthy as the cohorts of patients receiving monotherapy or combination therapy were very similar at baseline. Nevertheless, these findings should be interpreted with caution, as the data are observational and no randomization was involved. A treatment bias cannot be excluded, and it is possible that combination therapy was preferentially given to patients where comorbidity was felt less significant.

## Strengths and limitations

The strengths of this study include its high external (patient data derived from a multicentre, international registry) and internal validity (diagnosis of PAH by RHC). The risk of lead-time bias was minimized by including only incident patients. The low number of patients lost to follow-up provided robustness of data. We reported on all-cause mortality as the clinically most important and meaningful endpoint.

We acknowledge several limitations of our study. First, the presence of selection bias towards more severe patients due to participation of the tertiary centres must be assumed. Second, the number of patients in several subgroups was low and we were unable to perform analyses on possible associations between initial treatment and survival in CTD subgroups other than SSc. Third, we are unaware of the screening algorithm for PAH patients in the referring centres, which could theoretically cause a referral bias and influence the results of our study. However, clinical characteristics of the included patients did not indicate such a systematic bias. Fourth, missing values are unavoidable in an observational study. Fifth, SSc-PAH patients had a higher FVC and a higher FEV_1_ than SLE-PAH patients, which is an unexpected finding that cannot be explained with the available data. Finally, data concerning disease subtypes, classification criteria used for the different CTDs, longitudinal follow-up of UCTD patients and whether they developed into specific CTDs, specific CTD manifestations, short-term use of iloprost for peripheral vascular manifestations and immunosuppressive therapy was not captured in the COMPERA database.

## Supplementary Material

kead360_Supplementary_Data

## Data Availability

Primary data are available from Marius Hoeper upon reasonable request.
