# Supplementary material for: Treatment strategies and survival of patients with connective tissue disease and pulmonary arterial hypertension: a COMPERA analysis
Source: Rheumatology (Oxford). 2023 Jul 18;63(4):1139–46. doi: 10.1093/rheumatology/kead360 (PMC10986797; doi:10.1093/rheumatology/kead360)
Supplement: kead360_Supplementary_Data [file kead360_supplementary_data.docx]

# ONLINE SUPPLEMENTARY

| Supplementary Table S1. Diagnoses in the “other CTD-PAH” group | | |
| --- | --- | --- |
|  | **n** | **%** |
| Rheumatoid arthritis (RA) | 27 | 30.0 |
| Sjögren’s syndrome | 22 | 24.4 |
| Inflammatory myopathy | 12 | 13.3 |
| Overlap (fulfilling two classification criteria) | 12 | 13.3 |
| Other autoimmune rheumatic diseases | 17 | 18.9 |
| Morbus Bechterew | 4 | 4.4 |
| Microscopic polyangiitis | 2 | 2.2 |
| Polymyalgia rheumatica | 2 | 2.2 |
| Antiphospholipid syndrome; CTEPH | 1 | 1.1 |
| APAH | 1 | 1.1 |
| Autoimmune hepatitis | 1 | 1.1 |
| Giant cell arteritis | 1 | 1.1 |
| Morbus Still | 1 | 1.1 |
| Pauci-immune vasculitis | 1 | 1.1 |
| Psoriasis | 1 | 1.1 |
| Takayasu arteritis | 1 | 1.1 |
| Undifferentiated rheumatic disease | 1 | 1.1 |
| Total | 90 | 100.0 |

| Supplementary Table S2. Causes of death in the overall patient population | | | | | |
| --- | --- | --- | --- | --- | --- |
|  | **SSc (n=158)** | **SLE (n=8)** | **MCTD (n=9)** | **UCTD (n=22)** | **Other (n=31)** |
| Pulmonary hypertension / right heart failure | 75 (47.5) | 3 (37.5) | 7 (77.8) | 6 (27.3) | 15 (48.4) |
| Accident |  |  |  |  | 1 (3.2) |
| Age/ comorbidities | 1 (0.6) |  |  |  |  |
| Atrioventricular block | 1 (0.6) |  |  |  |  |
| Bowel ischemia; colitis | 1 (0.6) |  |  |  |  |
| Cancer, lung | 4 (2.5) | 1 (12.5) |  |  |  |
| Cancer, other | 3 (1.9) |  |  | 2 (9.1) | 1 (3.2) |
| Cardiac death | 5 (3.2) | 1 (12.5) |  | 3 (13.6) | 2 (6.5) |
| Cerebral death |  |  |  |  | 1 (3.2) |
| Cerebral ischemia; renal failure | 1 (0.6) |  |  |  |  |
| COPD | 1 (0.6) |  |  |  |  |
| COVID 19 |  |  |  |  | 1 (3.2) |
| CTD |  |  |  | 1 (4.6) |  |
| CTD-ILD | 1 (0.6) |  |  |  |  |
| Embolism, lung |  |  |  | 1 (4.5) |  |
| Haemorrhage |  |  | 1 (11.1) |  |  |
| Haemorrhage, cerebral |  |  |  |  | 1 (3.2) |
| Haemorrhage, gastrointestinal | 1 (0.6) |  |  |  |  |
| Haemorrhage, pulmonal |  |  |  |  | 1 (3.2) |
| Haematoma, subdural | 1 (0.6) |  |  |  |  |
| Hypoxemia | 1 (0.6) |  |  |  |  |
| Leukaemia | 1 (0.6) |  |  |  |  |
| Lung fibrosis | 1 (0.6) |  |  |  |  |
| Multi organ failure | 2 (1.3) |  |  |  |  |
| Palliative sedation | 1 (0.6) |  |  |  |  |
| Pneumonia | 3 (1.9) |  |  |  | 1 (3.2) |
| Renal failure | 2 (1.3) | 1 (12.5) |  | 1 (4.5) |  |
| Respiratory failure | 6 (3.8) |  |  | 3 (13.6) | 1 (3.2) |
| Sepsis; cardiogenic shock |  |  |  | 1 (4.5) |  |
| Sepsis; pneumonia |  |  |  |  | 1 (3.2) |
| Sepsis; pneumonia; renal failure |  |  |  |  | 1 (3.2) |
| Sepsis/ infection | 7 (4.4) |  | 1 (11.1) | 1 (4.5) | 1 (3.2) |
| Surgical complications | 1 (0.6) |  |  |  |  |
| Systemic sclerosis | 2 (1.3) |  |  |  |  |
| Unknown, sudden death | 2 (1.3) |  |  |  |  |
| Unknown | 34 (21.5) | 2 (25.0) |  | 3 (13.6) | 3 (9.7) |
| Values are n (%). | | | | | |

| Supplementary Table S3. Causes of death in patients with SSc-PAH according to ERA monotherapy, PDE5i monotherapy, or ERA + PDE5i dual therapy | | | |
| --- | --- | --- | --- |
|  | **ERA mono (n=60)** | **PDE5i mono (n=61)** | **ERA+PDE5i (n=19)** |
| Pulmonary hypertension / right heart failure | 30 (50.0) | 28 (45.9) | 8 (42.1) |
| Age/ comorbidities | 1 (1.7) |  |  |
| Bowel ischemia, colitis |  | 1 (1.6) |  |
| Cancer, lung | 1 (1.7) | 3 (4.9) |  |
| Cancer, other |  | 2 (3.3) |  |
| Cardiac death | 1 (1.7) | 1 (1.6) | 2 (10.5) |
| Cerebral ischemia, renal failure | 1 (1.7) |  |  |
| COPD | 1 (1.7) |  |  |
| CTD-ILD |  | 1 (1.6) |  |
| Haemorrhage, gastrointestinal | 1 (1.7) |  |  |
| Haematoma, subdural |  | 1 (1.6) |  |
| Hypoxemia |  | 1 (1.6) |  |
| Leukaemia |  | 1 (1.6) |  |
| Lung fibrosis |  |  | 1 (5.3) |
| Multiorgan failure | 1 (1.7) | 1 (1.6) |  |
| Palliative sedation | 1 (1.7) |  |  |
| Pneumonia | 1 (1.7) | 1 (1.6) |  |
| Renal failure | 2 (3.3) |  |  |
| Respiratory failure | 2 (3.3) | 4 (6.6) |  |
| Sepsis/ infection | 1 (1.7) | 3 (4.9) | 1 (5.3) |
| Surgical complications |  | 1 (1.6) |  |
| Systemic sclerosis | 1 (1.7) | 1 (1.6) |  |
| Unknown, sudden death | 1 (1.7) | 1 (1.6) |  |
| Unknown | 14 (23.3) | 10 (16.4) | 7 (36.8) |
| Values are n (%) | | | |

| Supplementary Table S4: Factors associated with survival in multivariable Cox regression | | |
| --- | --- | --- |
|  | **OR (95% CI)** | **P-value** |
|  |  |  |
| ERA mono (ref) | 1.00 |  |
| PDE5i mono | 0.89 (0.60;1.32) | 0.563 |
| ERA+PDE5i | 0.50 (0.29;0.87) | 0.014 |
| Age (per 10 yrs) | 1.19 (0.99;1.43) | 0.062 |
| WHO-FC IV* | 1.85 (1.09;3.15) | 0.022 |
| 6MWD (per 50 m) | 0.89 (0.80;1.00) | 0.049 |
| DLco (per 10%pred) | 0.82 (0.68;0.99) | 0.039 |
| Log_10_(NT-proBNP) | 1.89 (1.21;2.94) | 0.006 |
| * As WHO-FC III did not show differences to class I-II, class I-III were merged.  Shown are pooled results of those variables which were selected in >50% of the 10 imputed data sets. | | |
